# Prohibitin: an unexpected role in sex dimorphic functions

**DOI:** 10.1186/s13293-016-0083-9

**Published:** 2016-06-24

**Authors:** K. Hoa Nguyen, Sudharsana R. Ande, Suresh Mishra

**Affiliations:** Department of Internal Medicine, John Buhler Research Centre, University of Manitoba, Rm 843, 715 McDermot Avenue, Winnipeg, Manitoba R3E 3P4 Canada; Department of Physiology and Pathophysiology, University of Manitoba, Winnipeg, Canada

**Keywords:** Inflammation, Obesity, Insulin resistance, Metabolic dysregulation, Adipose-immune interaction, Pleiotropic proteins

## Abstract

Sex differences are known to exist in adipose and immune functions in the body, and sex steroid hormones are known to be involved in sexually dimorphic biological and pathological processes related to adipose-immune interaction. However, our knowledge of proteins that mediate such differences is poor. Two novel obese mice models, Mito-Ob and m-Mito-Ob, that have been reported recently have revealed an unexpected role of a pleiotropic protein, prohibitin (PHB), in sex differences in adipose and immune functions. This discovery points towards a role of pleiotropic proteins and their potential interplay with sex steroid hormones in mediating sexually dimorphic adipose-immune interaction.

Mitochondrial functions encompass the anaplerotic tricarboxylic acid cycle and energy production, but these functions are often finely tuned to serve specific roles in different cell types or tissues. Examples are lipogenesis in white adipocytes, thermogenesis in brown adipocytes, insulin secretion from pancreatic β cells, and cytokine production from immune cells. Thus, mitochondrial proteins involved in fundamental aspects of mitochondrial biology are uniquely placed to perform pleiotropic functions and provide an opportunity to simultaneously manipulate two different functions in two different cell types or tissues. However, to the best of our knowledge, a potential use of such pleiotropic protein in manipulating two different functions in two different cell types have not been discussed earlier.

It has been more than 25 years since McClung et al. [1] have discovered prohibitin (PHB, also known as PHB1) as an anti-proliferative gene. Up till now, the mechanism of anti-proliferative function of PHB remains controversial. Meanwhile, genetic studies with different model organisms have provided evidence for an important role of PHB as a scaffolding protein in mitochondrial biology [2], and emerging evidence suggests that PHB has a role in adipogenesis [3–5]. Furthermore, PHB has been reported to function as an adaptor protein in membrane-associated cell signaling functions in various cell types, including immune cells [6–8]. The plasma membrane-associated cell signaling function of PHB requires phosphorylation of PHB at different residues [6, 8]. One of them is tyrosine-114 in PHB protein [6]. However, it remains unclear whether mitochondrial and cell signaling functions are interrelated or independent from each other.

Recently, Ande et al. [9–11] have capitalized on the pleiotropic attribute of PHB to simultaneously manipulate adipocyte and immune cell-specific functions of PHB in transgenic mice models, Mito-Ob and m-Mito-Ob. This was achieved by expressing PHB (Mito-Ob) and m-PHB (m-Mito-Ob), a phosphomutant form of PHB lacking tyrosine-114 phosphorylation site, from the *aP2* gene promoter [10, 11]. Because the *aP2* gene is known to primarily expressed in adipocytes but also expressed in monocytic macrophages and dendritic cells among various immune cell types (e.g., T and B cells, NK cells, mast cells, plasma cells, neutrophils, basophils, and eosinophils) [12–14]. Overexpression of PHB in adipocytes was found to induce mitochondrial biogenesis, adipocyte hypertrophy, and increase in adipose tissue mass in both female and male transgenic mice compared with their age- and sex-matched wild-type littermates [10]. A similar effect of m-PHB on adipocytes was found in m-Mito-Ob mice suggesting that phosphorylation of tyrosine-114 is not required for mitochondria-related adipogenic function of PHB [11]. Of note, Mito-Ob and m-Mito-Ob mice were found to gain weight after puberty. Interestingly, the metabolic phenotype of Mito-Ob and m-Mito-Ob mice revealed a sex dimorphic role of PHB in adipocyte and immune cell functions as only male Mito-Ob/m-Mito-Ob mice developed obesity-associated adipose inflammation, impaired glucose homeostasis, and insulin resistance [9–11]. With aging, a sex-specific metabolic dysregulation in male Mito-Ob mice led to the development of nonalcoholic steatohepatitis (NASH) and hepatocellular carcinoma (HCC), suggesting sex differences in adipose-hepatic crosstalk in Mito-Ob mice [15]. Intriguingly, metabolic dysregulation in male m-Mito-Ob mice led to sinus histiocytosis with massive lymphadenopathy (SHML), revealing an anti-proliferative role of tyrosine-114 in monocytic macrophages and dendritic cells. Female Mito-Ob and m-Mito-Ob remained protected from HCC and SHML, respectively, despite comparable obesity with their male counterparts [10, 11, 15]. Intriguingly, on a high-fat diet, male m-Mito-Ob mice develop adult-onset type 1 diabetes instead of tumors [16]. The development of HCC and SHML or autoimmune diabetes in a male sex-specific manner suggests a sex dimorphic effect of PHB on adipocyte, monocytic macrophage, and dendritic cell functions, which were not suspected before [17].

Similar to Mito-Ob and m-Mito-Ob mice, an increase in body weight after puberty has been observed in the female heterozygous *Phb2* knockout mice [18]. In this context, it is important to note that PHB and its homologous protein PHB2 heterodimerizes in the inner mitochondrial membrane and play a role in the maintenance of mitochondrial structure and functions [2]. In addition, PHB2 also functions as a repressor for estrogen activity [19]. Moreover, PHB has been identified as a target gene for estrogen [20]. These evidences suggest that the relationship between PHB and estrogen is complex and both may regulate each other’s function in the regulation of adipose tissue homeostasis [17].

HCC is sexually dimorphic in humans and rodents with higher prevalence in males, an effect that depends on sex steroids [21, 22]. The molecular mechanisms by which estrogens prevent and androgens promote liver cancer remain unclear. The focus has been on direct effect of sex steroids on hepatocytes; however, a potential role of other cell or tissue type is not explored. Recently, Foxa1/2 has been shown to play an important role in sexual dimorphism of HCC as revealed by reversal of sexually dimorphic HCC in Foxa1/2-deficient mice after DEN-induced hepatocarcinogenesis [21]. At the same time, the authors also noticed that without carcinogen treatment, Foxa1/2 mutant mice maintain much of the sexual dimorphism gene expression profile that is present in control livers [21]. This suggests that the loss of gender specificity in Foxa1/2-deficient mice occurs with the onset of carcinogen exposures. Given the fact that multiple physiological and pathophysiological processes are sexually dimorphic in the organs expressing Foxa1/2 proteins, it is likely that other stresses, like inflammatory changes, might also invoke gender-specific responses. Similarly, sex differences in HCC development in estrogen and androgen receptor knockout animals, although diminished, were not entirely abolished [23, 24]. Collectively, these findings suggest that factors other than sex steroids and tissues other than hepatic tissue also contribute to sex differences in the development of HCC [15, 17]. The Mito-Ob mice provide evidence that dysregulation of sex differences in adipocytes and immune cells can cause sex differences in HCC incidence. This makes sense because adipose, immune, and liver functions are intimately linked in systemic metabolic regulation, and their dysregulation is linked to a number of diseases. Of note, sex difference in HCC incidence in Mito-Ob mice is further extended to sex-specific HCC development in male and full protection in female. In this context, it is important to note that liver-specific PHB knockout mice have been reported earlier, which also develop HCC. In PHB knockout mice, out of 38 % (5/13 mice) mice that develop tumors between ages of 35 and 46 weeks, most were females (4/8 in females and 1/5 in males) [25]. This would imply that in PHB knockout mice, sex dimorphism in HCC occurrence is reversed with increased susceptibility in females compared to male counterpart. Taken together, these findings suggest that PHB overexpression further polarizes sex differences while its knockdown reverses the effect on sex differences in adipose, immune, and hepatic functions. Collectively, these evidences suggest that PHB has an important role in mediating sex differences in a number of cell or tissue types. Whether PHB mediates such effects as a downstream target of sex steroids or independent of sex steroids remains to be determined, which warrants further investigation. The development of SHML in the ovariectomized female m-Mito-Ob mice suggests a role of sex steroid in the modulation of PHB function in adipocyte and immune cell functions [11].

In summary, the discovery of PHB in mediating sex differences and adipose and immune functions and consequently in adipose-immune interaction raises important questions related to the role of PHB in the biology of sex differences such as the following:

1. What is the mechanism of PHB-induced sex differences in adipocyte and immune cell functions?
2. Does PHB mediate sex-specific functions?
3. What is relationship between PHB and sex steroids in mediating sex dimorphic functions?

It is anticipated that a better understanding of the role of PHB in sexually dimorphic functions in the body may lead to the discovery of novel ways to modulate adipose and immune functions and contribute to the development of more effective, gender-responsive, and personalized medicine.
